# The Induction of an Effective dsRNA-Mediated Resistance Against Tomato Spotted Wilt Virus by Exogenous Application of Double-Stranded RNA Largely Depends on the Selection of the Viral RNA Target Region

**DOI:** 10.3389/fpls.2020.533338

**Published:** 2020-11-26

**Authors:** Saeid Tabein, Marco Jansen, Emanuela Noris, Anna Maria Vaira, Daniele Marian, S. Ali Akbar Behjatnia, Gian Paolo Accotto, Laura Miozzi

**Affiliations:** ^1^Department of Plant Protection, Faculty of Agriculture, Shahid Chamran University of Ahvaz, Ahvaz, Iran; ^2^Plant Virology Research Center, College of Agriculture, Shiraz University, Shiraz, Iran; ^3^Institute for Sustainable Plant Protection, National Research Council of Italy, Turin, Italy; ^4^Laboratory of Virology, Department of Plant Sciences, Wageningen University & Research, Wageningen, Netherlands

**Keywords:** RNAi-based vaccination, double-stranded rnas, orthotospovirus, ambisense RNA, nucleocapsid protein, cell-to-cell movement protein

## Abstract

Tomato spotted wilt virus (TSWV) is a devastating plant pathogen, causing huge crop losses worldwide. Unfortunately, due to its wide host range and emergence of resistance breaking strains, its management is challenging. Up to now, resistance to TSWV infection based on RNA interference (RNAi) has been achieved only in transgenic plants expressing parts of the viral genome or artificial microRNAs targeting it. Exogenous application of double-stranded RNAs (dsRNAs) for inducing virus resistance in plants, namely RNAi-based vaccination, represents an attractive and promising alternative, already shown to be effective against different positive-sense RNA viruses and viroids. In the present study, the protection efficacy of exogenous application of dsRNAs targeting the nucleocapsid (*N*) or the movement protein (*NSm*) coding genes of the negative-sense RNA virus TSWV was evaluated in *Nicotiana benthamiana* as model plant and in tomato as economically important crop. Most of the plants treated with *N*-targeting dsRNAs, but not with *NSm*-targeting dsRNAs, remained asymptomatic until 40 (*N. benthamiana*) and 63 (tomato) dpi, while the remaining ones showed a significant delay in systemic symptoms appearance. The different efficacy of *N*- and *NSm*-targeting dsRNAs in protecting plants is discussed in the light of their processing, mobility and biological role. These results indicate that the RNAi-based vaccination is effective also against negative-sense RNA viruses but emphasize that the choice of the target viral sequence in designing RNAi-based vaccines is crucial for its success.

## Introduction

Tomato spotted wilt virus (TSWV), genus *Orthotospovirus*, family *Tospoviridae*, belongs to the list of the ten most economically important viruses in the world (Scholthof et al., 2011) and is able to cause high yield losses in a variety of crops and ornamentals, in tropical and subtropical regions (Pappu et al., 2009; Mitter et al., 2013; Turina et al., 2016). It has a wide host range and is transmitted by thrips in a persistent manner (Whitfield and Rotenberg, 2015). The TSWV particles are spherical and surrounded by a host-derived membrane with a diameter of 80–120 nm. The TSWV genome is of negative/ambisense polarity and consists of three linear single-stranded RNAs (ssRNA) named large (L), medium (M), and small (S) according to their sizes (Turina et al., 2016). The L segment codes for the RNA-dependent RNA polymerase (RdRp) in negative sense, while M and S segments are ambisense in their genome organization; the M RNA encodes the glycoprotein precursor (GP) of the mature membrane glycoproteins G_N_ and G_C_ and the cell-to-cell movement protein (NSm) while the S RNA codes for the nucleocapsid protein (N), and the silencing suppressor protein (NSs).

Up to now, few resistance genes against TSWV have been identified and introgressed in commercial cultivars (Turina et al., 2016); however, the frequent appearance of resistance-breaking isolates, together with the difficulty of selecting and incorporating new resistance genes stress the needs of developing new strategies for protecting plants against TSWV infection.

RNA interference (RNAi) is an RNA-mediated regulatory mechanism, conserved in most eukaryotes, consisting in the sequence-specific degradation of target RNA guided by the complementary small RNAs (sRNAs; Meister and Tuschl, 2004). Beside its crucial activity in regulating growth and development, this mechanism plays a critical role in host defense against subcellular pathogens (Baulcombe, 2004; Padmanabhan et al., 2009; Agius et al., 2012). RNAi is triggered by double-stranded RNAs (dsRNA) or hairpin RNAs (hpRNA) that are processed by specific nucleases called DICER or DICER-LIKE (DCL) into sRNAs able to guide the cleavage of complementary single-stranded RNAs, such as messenger RNAs or viral genomic/antigenomic RNAs (Meister and Tuschl, 2004).

Virus infection is associated with the accumulation of viral small RNAs (vsRNAs), originated by the host plant RNAi machinery from viral dsRNAs or hpRNAs. VsRNAs are able to direct the degradation of complementary viral single-stranded RNAs through the plant RNAi pathways (Agius et al., 2012). As a consequence, viruses are both inducers and targets of RNAi, through a process regarded as a natural antiviral defense mechanism in plants (Guo et al., 2016). Since the discovery of such siRNA-mediated antiviral defense mechanism, a number of transgene- or virus-based silencing technologies have been developed in order to protect plants from pathogens (Dietzgen and Mitter, 2006; Guo et al., 2016). DsRNAs or hpRNAs transcribed from engineered inverted repeats were shown to be potent inducers of a gene silencing response when directed against transgenes or viral pathogens (Smith et al., 2000; Marjanac et al., 2009; Hameed et al., 2017). Different RNAi approaches, all based on plant transformation, were established to induce resistance against TSWV, including the expression of viral genomic sequences (Yazhisai et al., 2015), artificial microRNAs (miRNAs; Mitter et al., 2016), or synthetic *trans*-acting siRNAs (tasiRNAs; Carbonell et al., 2019). However, transgenic approaches are time-consuming, expensive and require efficient plant transformation protocols; moreover, they are prone to significant regulation and acceptance issues, particularly in the case of food crops.

A promising technique is the RNAi-based vaccination, that exploits the RNAi machinery of the plants to protect them from viral infection. Several studies have shown that dsRNAs homologous to viral sequences, when topically applied to plants, can induce RNAi-mediated defense, interfering with infection by plant positive RNA viruses and viroids in a sequence-specific manner [reviewed in Mitter et al. (2017b); Dubrovina and Kiselev (2019))]. Exogenous application of naked dsRNAs (Tenllado and Díaz-Ruíz, 2001; Tenllado et al., 2004; Petrov et al., 2015) or spraying of bacterially expressed dsRNAs (Tenllado et al., 2003; Gan et al., 2010) were demonstrated to protect plants up to 5 days post treatment. Moreover, loading dsRNAs onto clay nanosheets increased dsRNA stability and efficacy up to 20 days post spray (Mitter et al., 2017a). These approaches interfered with the infection of potyviruses, bromoviruses, tobamoviruses, and potexviruses (Tenllado and Díaz-Ruíz, 2001; Tenllado et al., 2004; Petrov et al., 2015; Mitter et al., 2017b).

In the present study, we tested the protective effect of exogenous application of dsRNAs against TSWV, a negative sense RNA virus, belonging to the *Tospoviridae* family, both in the model plant *Nicotiana benthamiana* and in tomato (*Solanum lycopersicum* L.), one of the most important horticultural crops worldwide. For this purpose, we selected two genomic regions, one covering the N gene, which encodes a structural protein, and another covering the NSm gene encoding a non-structural protein. The dsRNAs were synthesized *in vitro* and applied on plant leaves that were further inoculated with TSWV. Plants were monitored for local and systemic symptom development and for the presence of TSWV. Persistence and movement of the dsRNAs in plants were also studied.

## Materials and Methods

### Biological Material

*Nicotiana benthamiana* and tomato plants were maintained in a growth chamber at 24°C with a light/dark cycle of 16/8 h. Plants with 4–5 fully expanded leaves were used for the bioassays. Plants were inoculated with a TSWV pepper isolate [P105, PLAVIT collection, IPSP-CNR Torino, Italy, World Data Center for Microorganism (WDCM) no. 1057, http://www.wfcc.info/ccinfo/collection/col_by_country/i/39/].

### *In vitro* dsRNA Synthesis

To produce dsRNA molecules, a two-step PCR approach followed by *in vitro* transcription was used (Voloudakis et al., 2015). In the case of *N*- and *NSm*-targeting dsRNAs, total RNA from TSWV-infected *N. benthamiana* plants was extracted using Trizol (Life Technologies, United States) according to manufacturer’s instructions. 1 μg of total RNA extracted from TSWV-infected *N. benthamiana* plants was used as template for cDNA synthesis, using the High-Capacity cDNA Reverse Transcription Kit (Thermo Fisher Scientific, Waltham, MA, United States), according to manufacturer’s instructions. Viral fragments were amplified with specific primers designed using Primer3 software^1^. The T7 RNA polymerase promoter/binding site sequence was added at the 5 ì end of both the forward and the reverse primers (Supplementary Table 1). PCR was carried out in a final volume of 50 μl, containing 10X reaction buffer, 1 μl cDNA template (diluted 1:5), 200 μM dNTPs, 0.2 μM of each primer, 1.5 mM MgCl_2_, and 2.0 units of Platinum *Taq* DNA polymerase (Invitrogen, Thermo Fisher Scientific, Waltham, MA, United States). After an initial denaturation at 95°C for 10 min, the first 10 cycles were performed as follows: 95°C for 30 s, 40°C for 45 s, 72°C for 1 min, followed by 35 cycles each of 95°C for 30 s, 55°C for 45 s, and 72°C for 1 min. After a final extension at 72°C for 7 min, PCR fragments were loaded on 1% agarose gel and purified by the DNA Clean and Concentrator kit (Zymo Research, Irvine, United States). To *in vitro* synthesize sense and antisense viral derived ssRNAs, 1 μg of purified DNA template was used in 50 μl transcription reaction containing T7 reaction buffer, 500 μM rNTPs, 5 mM DTT, 50 units of T7 RNA polymerase (Invitrogen, Thermo Fisher Scientific, Waltham, MA, United States), conducted at 37°C for 2 h. After removing DNA template by TURBO RNase-free DNAse (Ambion, Thermo Fisher Scientific, Waltham, MA, United States), dsRNAs were obtained by mixing the specific sense and antisense ssRNAs, then incubating at 95°C for 3 min, and at 37°C for 30 min. The formation of dsRNAs was confirmed by treatment with Mung Bean Nuclease (New England Biolabs, MA, United States).

As negative control, a dsRNA targeting the replication initiator protein (Rep) coding region of the geminivirus *tomato yellow leaf curl Sardinia virus* (TYLCSV) was used. In this case, DNA was extracted from TYLCSV-infected *N. benthamiana* plants using the TLES method described in Maffei et al. (2014). The *Rep*-targeting dsRNA was synthetized as described above, using specific primers reported in Supplementary Table 1.

### Virus Inoculation and dsRNAs Exogenous Application

In order to ensure the uniformity of the TSWV inoculum in all the experiments, a TSWV inoculum stock was prepared from systemically infected *N. benthamiana* leaves. For this, symptomatic leaf tissue was cut in small slices, split into 500 mg aliquots and stored in liquid nitrogen until use. Inoculation was performed by homogenizing each aliquot in 50 ml of inoculation buffer (5 mM diethyldithiocarbamic acid, 1 mM ethylenediaminetetraacetic acid, and 5 mM sodium sulfite), and applying a 50 μl-dose to the upper side of two different leaves of each plant previously dusted with carborundum. When required, 10 μg of dsRNAs were added to the inoculum, just before inoculation.

*Nicotiana benthamiana* plants were used to evaluate the effect of dsRNAs on local lesions. Systemic infection induced by TSWV was evaluated in *N. benthamiana* and tomato plants, at different time points from the inoculation. Plants were inoculated with TSWV together with dsRNAs (*N* and *NSm*). The positive control group was inoculated with TSWV only and water. As negative controls, one group of plants received TSWV and the *Rep*-targeting dsRNAs originated from TYLCSV, while another one received only water. A total of thirteen *N. benthamiana* and seven tomato plants per treatment were analyzed in three and one different experiments, respectively. Symptoms development was monitored until 40 days post inoculation (dpi) in the case of *N. benthamiana* and 63 dpi in the case of tomato. Presence of viral RNA was checked by PCR in newly emerged leaves that did not receive the inoculum.

### *In planta* dsRNA Movement

In order to evaluate the persistence/movement of dsRNAs in the plants that were not inoculated with TSWV, 10 μg of dsRNAs were mixed with 100 μl of sterile water and mechanically inoculated on two carborundum-dusted fully expanded leaves (50 μl per leaf) by gentle rubbing. Two leaf disks from each treated (local) and first expanded leaf from the apex (systemic) were collected at 1, 3, 6, and 9 dpi. Three plants were used for each time point. Just before sampling, leaves were washed with Triton X-100 (0.05%) and water to eliminate residual dsRNAs present on the leaf surface. Total RNA was extracted by Trizol reagent (Life Technologies, United States). cDNA synthesis was performed with random primers using the High-Capacity cDNA Reverse Transcription Kit (Thermo Fisher Scientific, Waltham, MA, United States), according to manufacturer’s instructions.

### Detection of TSWV and dsRNAs by RT-PCR

Tomato spotted wilt virus infection was evaluated by RT-PCR with a primers pair (TSWV_L_fw/TSWV_L_rv, amplicon size 646 bp; Supplementary Table 1) designed on the L RNA, a genomic component unrelated to the N and NSm genes used to design the dsRNAs. In the dsRNAs mobility experiment, the presence of *N*- and *NSm*-targeting dsRNAs was estimated by PCR using primers TSWV_N_fw/TSWV_N_rv and TSWV_NSm_fw/TSWV_NSm_rv, respectively (Supplementary Table 1). PCR was performed in a final volume of 25 μl, containing 2.5 μl reaction buffer, 1 μl cDNA template, 200 μM each dNTPs, 0.4 μM each primer, 2 mM MgCl2, and 1 unit Platinum *Taq* DNA polymerase (Invitrogen). After an initial denaturation step for 4 min at 95°C, 30 cycles consisting each of 30 s at 95°C, 30 s at 58°C, and 30 s at 72°C were performed, followed by a final extension step for 7 min at 72°C.

### Detection of dsRNAs by Quantitative RT- PCR

Quantitative RT-PCR (qRT-PCR) was carried out using iCycler iQTM Real-Time PCR Detection System (BioRad Laboratories, Hercules, CA, United States), with the following cycling parameters: 1 cycle at 95°C for 5 min, 45 cycles, each consisting of 15 s at 95°C and 1 min at 60°C. A melting curve was recorded at the end of each run to assess the specificity of amplification. All reactions were performed with three technical replicates. RT-PCR efficiency was calculated using standard curves constructed with serial dilutions of cDNA extracted from infected plants. Data acquisition and analysis were handled by the BioRad iCycler software (version 3.06070) that calculates Ct values and standard curves. qRT_TSWV_N_fw/qRT_TSWV_N_rv (Mason et al., 2002) and qRT_TSWV_NSm_fw/qRT_TSWV_NSm_rv primers pairs (Supplementary Table 1) were used to amplify exogenous dsRNAs, while the primer pair qRT_NbCOX_fw/qRT_NbCOX_rv (Supplementary Table 1) was used for the amplification of the *N. benthamiana* gene Niben101Scf02399 coding for the cytochrome c oxidase (NbCOX), used as reference gene (Nerva et al., 2017). The relative dsRNAs amount was estimated for each sample using the ΔCt method, where ΔCt is |Ct_dsRNA_ – Ct_cox_|.

### Small RNA Analysis by High Throughput Sequencing (HTS)

Small RNA populations originating from *N*- and *NSm*-targeting dsRNAs were analyzed in samples collected for the dsRNA movement investigation at 1 dpi. Small RNA libraries preparation and sequencing were performed by Novogene (United Kingdom) Company Limited (United Kingdom). After adapter removal with fastp (Chen et al., 2018) and low-quality filtering and artifact removal with fastx-toolkit^2^, clean reads were mapped to the TSWV genome segment targeted by the applied exogenous dsRNA (S segment, Acc. Num. DQ376178.1; M segment, Acc. Num. KJ575621.1) using bowtie v1-3-0 (Langmead et al., 2009), with 0 mismatches. Mapping results were visualized using Misis (Seguin et al., 2014). Sequence data have been submitted to the Sequence Read Archive (SRA) with the BioProject ID PRJNA672300.

## Results

### Selection of Viral Genomic Sequences for dsRNA Production

In order to select the genomic regions of TSWV most suitable as target by dsRNAs, we first considered the vsRNAs profile of TSWV available in the literature (Mitter et al., 2013; Margaria et al., 2015), starting from the hypothesis that the genomic regions characterized by a high number of mapping vsRNAs could be more subjected to RNAi-mediated degradation. According to Mitter et al. (2013), most vsRNAs detected in TSWV-infected *N. benthamiana* plants mapped to the M segment, followed by the S segment, while the L RNA had the least number of mapping vsRNAs. Therefore, we focused our attention on the M and S segments. Among the two open reading frames (ORFs) of the M segment, *GP* and *NSm*, the *GP* ORF has the higher number of mapping vsRNAs (Mitter et al., 2013). However, we decided to select the *NSm* coding region as dsRNA target, since the movement protein has an important effect on viral systemic spread while the glycoproteins are not needed for *in planta* replication and intercellular spread of the virus; indeed, amiRNAs targeting the *GP* fragment did not show any antiviral protective effect (Carbonell et al., 2019). In the case of the S segment containing the *N* and *NSs* ORFs, we focused on the *N* coding region, since it codes for the nucleocapsid protein, a structural protein essential for virions formation. In addition, artificial miRNAs targeting the *NSs* ORF were not effective in protecting plants against TSWV infection (Mitter et al., 2016; Carbonell et al., 2019).

Since previous work showed that the exogenous dsRNAs must be longer than 300 bp to effectively interfere with virus infection (Tenllado and Díaz-Ruíz, 2001), we designed to produce dsRNAs longer than this threshold, i.e., 761 bp for the *N*-targeting dsRNA and 603 bp for the *NSm*-targeting dsRNA.

Finally, to better evaluate the dsRNAs protective effect, we decided to include a dsRNA homologous to a portion of the gene encoding the Rep pf TYLCSV as a negative control. The length of the *Rep*-targeting dsRNA (598 bp) was similar to the *N*- and *NSm*-targeting dsRNAs, but, due to the lack of homology with the TSWV genome, this dsRNA was not expected to show any protective effect against TSWV.

### Production of dsRNAs

**FIGURE 1:**
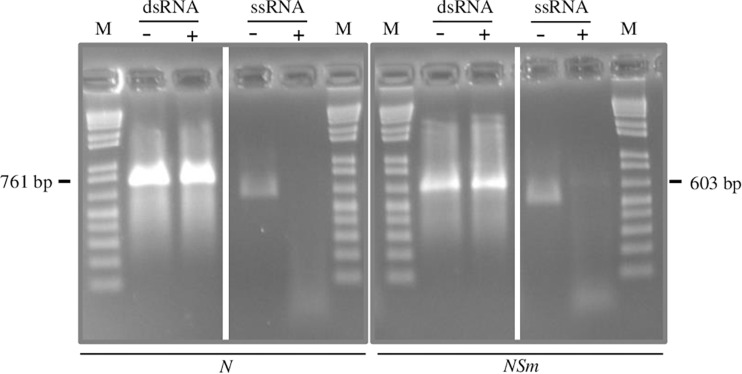
Confirmation of double-strand structure of *N*- and *NSm*-targeting dsRNAs by specific degradation with Mung Bean Nuclease. A single-stranded RNA transcript is used as control. +, Mung Bean Nuclease treatment; -, mock treatment; and M, 100 bp DNA Ladder (New England Biolabs, MA, United States). Nucleotide sequence length of synthetized fragments is shown on the sides.

The method employed to produce dsRNAs, based on two sequential PCR reactions, coupled with *in vitro* transcription (Voloudakis et al., 2015) typically yielded 50–80 μg of dsRNAs, starting from 1 μg of DNA template. The double-stranded nature of the molecules obtained was confirmed by incubating the *in vitro*-transcribed RNAs before and after the annealing step with Mung Bean Nuclease, an enzyme that specifically degrades single-stranded nucleic acids (Figure 1).

### Different Antiviral Efficacy of Exogenous Application of dsRNAs Targeting the *N* or *NSm* Genomic Regions of TSWV

**FIGURE 2:**
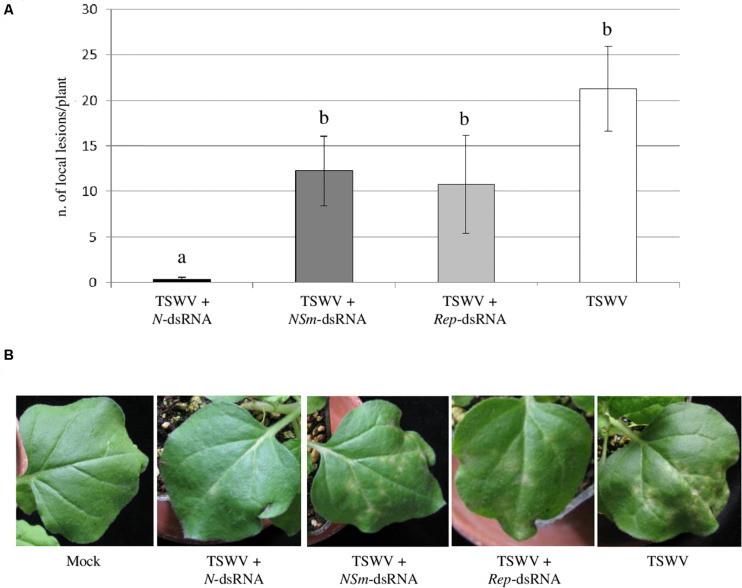
Effect of dsRNAs on production of TSWV-induced local lesions on *N. benthamiana* leaves. **(A)** Histogram reporting the number of local lesions per plant. Results are expressed as mean values of local lesions per plant counted at 7 dpi on 2 inoculated leaves for each plant, for a total of 13 plants per treatment, except for *Rep*-targeting dsRNAs treated plants for which 4 plants were considered. Different letters indicate significant differences at *P* < 0.05 (Kruskal–Wallis test followed by *post hoc* Wilcoxon test). **(B)** Local lesions at 7 dpi on *N. benthamiana* plants treated with *N*-, *NSm*- and *Rep*-targeting dsRNAs and inoculated with TSWV or inoculated with TSWV alone. Different letters indicate statistically significant differences.

At 7 dpi, typical chlorotic and necrotic local lesions occurred on leaves of *N. benthamiana* plants inoculated with TSWV alone, with no significant difference (*p*-value < 0.05) in their average number compared to plants inoculated with TSWV + *NSm*-targeting dsRNAs and with TSWV + *Rep*-targeting dsRNA. At the same time point, plants inoculated with TSWV + *N*-targeting dsRNAs showed drastic reduction in the number of local lesions (Figure 2), highlighting a protective effect of the *N*-targeting dsRNAs.

**FIGURE 3:**
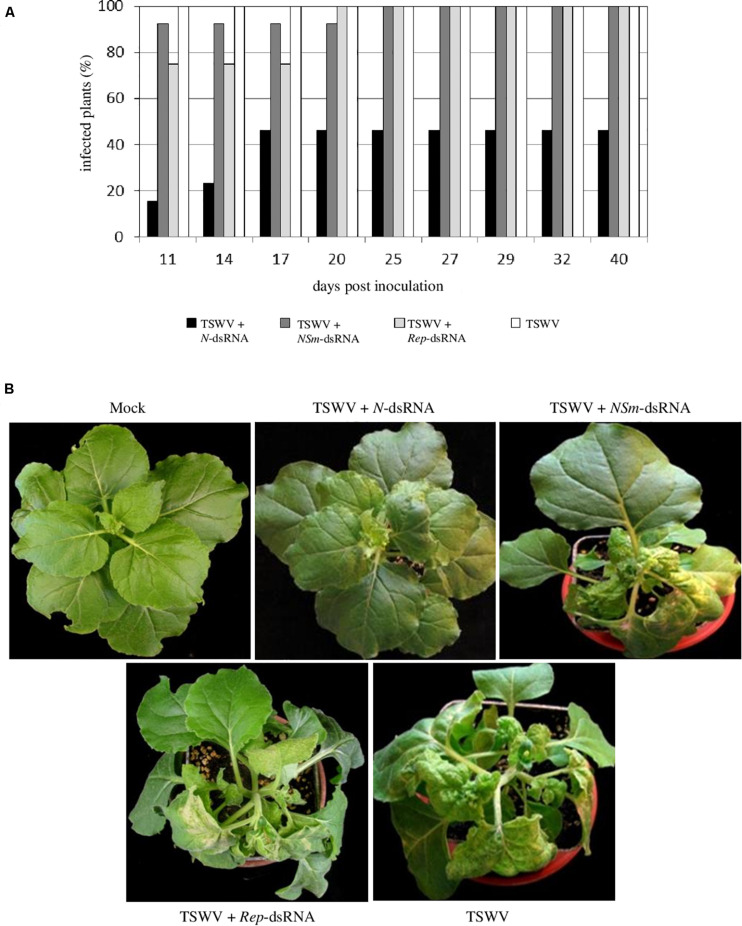
Efficacy of *N*-, *NSm*- and *Rep*-targeting dsRNA treatments against TSWV infection in *N. benthamiana*. **(A)** Percentage of TSWV-infected plants treated or not with dsRNAs. **(B)** Systemic symptoms at 14 dpi on *N. benthamiana* plants treated with *N*-, *NSm*- and *REP*-targeting dsRNAs and inoculated with TSWV or inoculated with TSWV alone.

All the *N. benthamiana* plants inoculated with TSWV alone showed typical systemic symptoms of chlorosis, necrosis, stunting, and wilting at 11 dpi. At the same time point, similar symptoms were observed on 92% of plants treated with TSWV + *NSm*-targeting dsRNAs and 75% of plants treated with TSWV + *Rep*-targeting dsRNAs; conversely, only 15% of plants treated with TSWV + *N*-targeting dsRNAs appeared symptomatic. At later observation (25 dpi), all plants treated with TSWV + *NSm*-targeting dsRNAs or with TSWV + *Rep*-targeting dsRNAs showed the systemic TSWV symptoms. In the case of plants treated with TSWV + *N*-targeting dsRNAs, the rate of symptomatic plants reached 46% at 17 dpi and remained unchanged until the end of the experiment (40 dpi; Figure 3). The RT-PCR performed on a subset of *N. benthamiana* plants confirmed visual symptoms evaluation (not shown).

**FIGURE 4:**
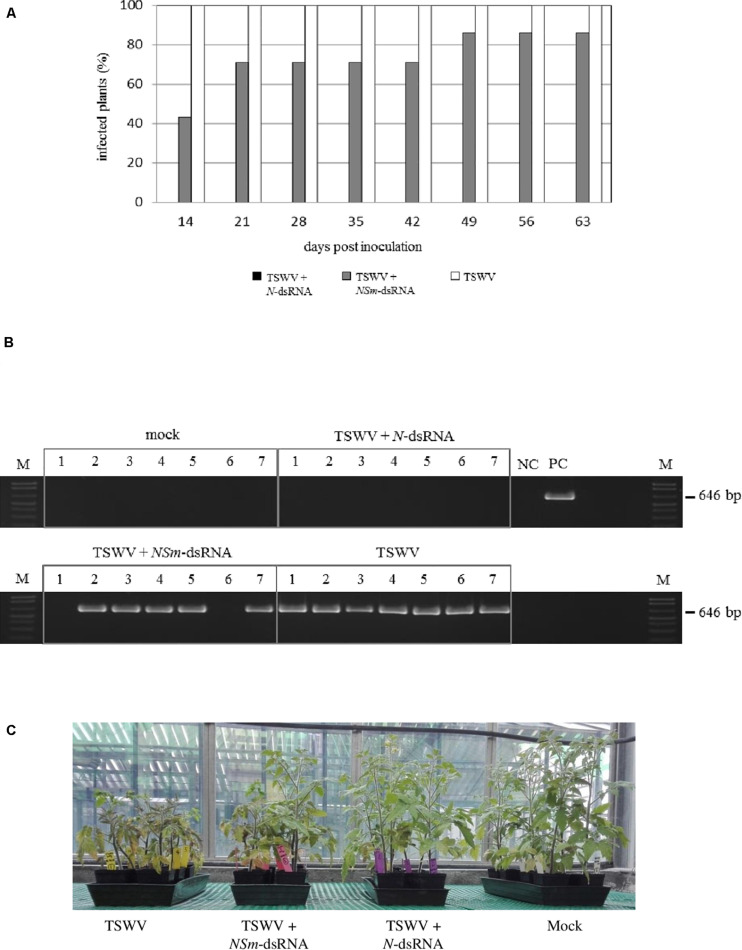
Efficacy of *N*- and *NSm*-targeting dsRNA treatments against TSWV infection in tomato; seven plants (1–7) were used for each thesis. **(A)** Percentage of TSWV infection in plants treated or not with dsRNAs at several time points. **(B)** RT-PCR for the evaluation of TSWV infection in tomato plants at 28 dpi; primers targeting TSWV L genomic segment were used. NC, negative control (Mock-inoculated plant); PC, positive control (TSWV-infected plant); and M, 100 bp DNA Ladder (New England Biolabs, MA, United States). Size of amplified fragment is shown on the side. **(C)** Tomato plants treated with *N*- and *NSm*-targeting dsRNAs and inoculated with TSWV or inoculated with TSWV alone, at 30 dpi.

In the case of tomato plants, most of the plants (71%) treated with TSWV + *NSm*-targeting dsRNAs and 100% of the plants inoculated only with TSWV were already infected at 21 dpi, but none of the plants inoculated with TSWV and treated with *N*-targeting dsRNAs became infected for the entire duration of the experiment, at 64 dpi (Figure 4A).

In both species, we didn’t observe any difference in the reproductive stage; all the asymptomatic plants, including those treated with dsRNAs, exhibited a normal flowering, while no flowering was observed in symptomatic plants, due to the heavy symptomatology.

The virus was detected in young leaves of all the symptomatic plants while it was absent in all asymptomatic plants treated with TSWV + *N*-targeting dsRNAs (Figure 4B), confirming visual inspection (Figure 4C).

Taken together, these results show that exogenous application of dsRNAs homologous to the *N* gene can completely suppress or robustly contrast viral replication and protects plants from TSWV infection, both in *N. benthamiana* and tomato, by inhibiting or strongly delaying symptom development, while dsRNAs targeting the *NSm* gene have almost no protective effect.

### Systemic Movement of the Applied dsRNAs

**FIGURE 5:**
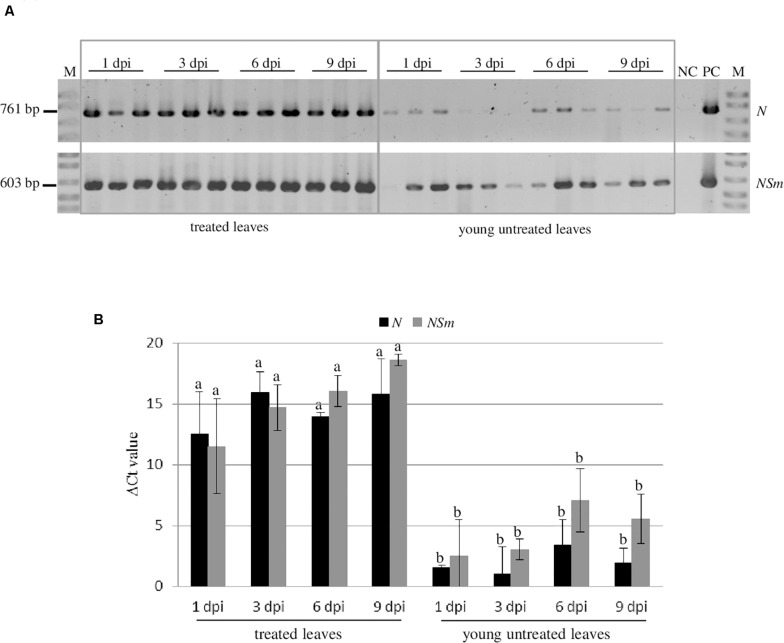
Persistence and systemic movement of *N*- and *NSm*-targeting dsRNAs. Three biological replicates were used. **(A)** Detection of the *N*- and *NSm*-targeting dsRNAs in treated leaves and untreated young leaves *of N. benthamiana* at different time points by end-point RT-PCR. NC, negative control (Mock-inoculated plant); PC, positive control (TSWV-infected plant); and M, 100 bp DNA Ladder (New England Biolabs, MA, United States). Sizes of amplified fragments are shown on the side. **(B)** Quantification of *N*- and *NSm*-targeting dsRNAs by qRT-PCR. The Ct values obtained for the dsRNAs were normalized with the Ct values obtained for the COX transcript used as reference. Vertical lines on each bar represent standard deviations. Different letters indicate statistically significant differences (*p* < 0.05, ANOVA). Different letters indicate statistically significant differences.

In order to investigate the persistence and the systemic movement of *N* and *NSm*-targeting dsRNAs applied on the leaves, in the absence of virus infection, we analyzed by RT-PCR the presence of both dsRNAs at 1, 3, 6, and 9 dpi in both treated (local persistence) or young untreated leaves (systemic movement) of *N. benthamiana* plants (Figure 5A). Both dsRNAs could be detected until 9 dpi in the treated leaves indicating that they persist there for several days after application. Moreover, we detected both *N* and *NSm*-targeting dsRNAs also in untreated leaves at all time points, although with a lower intensity compared to treated tissues.

When the quantitative evaluation of the dsRNAs was performed by qRT-PCR, we observed that most of the applied *N*- or *NSm*-targeting dsRNAs remained in the treated leaves and only a limited amount of them was transported systemically to the young untreated leaves. Interestingly, the dsRNAs systemic movement, even if very limited, could be observed at 1 dpi and remained steadily low until 9 dpi (Figure 5B).

These results indicate that both dsRNAs considered are able to move systemically within the plant, but that the majority of them remains in treated leaves. No significant differences in persistence or systemic movement capacity were observed between the *N*- and *NSm*-targeting dsRNAs.

### Analysis of the sRNAs Populations Originating From *N*- and *NSm*-Targeting dsRNAs

**FIGURE 6:**
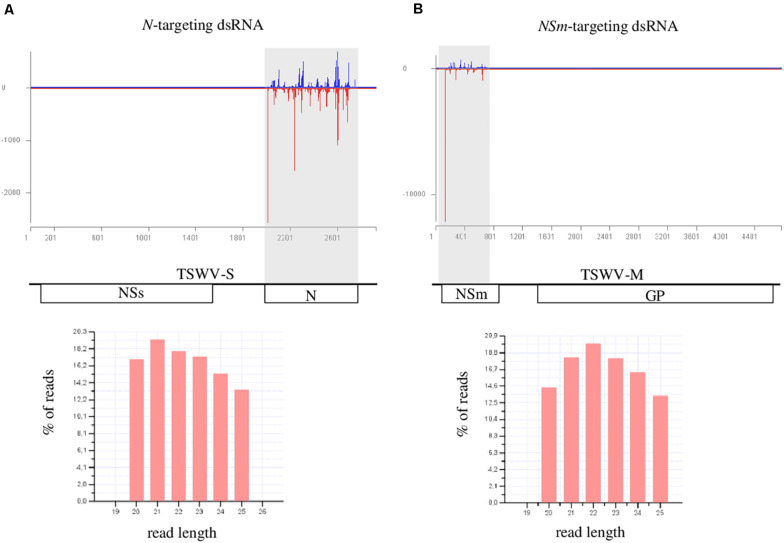
HTS analysis of small RNAs in dsRNAs-treated *N. benthamiana* leaves, 1 day post treatment. **(A)** upper panel: distribution of TSWV-targeted small RNAs along the S segment of TSWV genome (TSWV-S; Genbank acc. N. DQ376178.1); viral sense and antisense small RNAs are reported in blue and red, respectively; *N* (nucleoprotein); and *NSs* (non-structural protein) genes are represented along the viral RNA S segment; lower panel: length distribution of the small RNAs reads mapping to the TSWV S segment. **(B)** upper panel: distribution of TSWV-targeted small RNAs along the M segment of TSWV genome (TSWV-M; Genbank acc. N. KJ575621.1); NSm (non-structural protein); and GP (glycoproteins precursor) genes are represented along the viral RNA M segment; lower panel: length distribution of the small RNAs reads mapping to the TSWV M segment.

In order to evaluate if *N*- and *NSm*-targeting dsRNAs were processed by Dicer and were able to originate the silencing effector molecules (siRNAs), we sequenced the sRNAs populations of treated (local), and newly emerged non-treated (systemic) *N. benthamiana* leaves, 1 day post exogenous dsRNAs application. After adapter removal and quality filtering, 18 to 28 million of reads were obtained (Supplementary Tables 2, 3). Sequences with length ranging from 20 to 25 nt were selected for further analyses. In the case of leaves treated with dsRNAs (local), the percentage of reads mapping to the *N*- and *NSm*-targeting dsRNAs regions was 0.39 and 0.50, respectively (Figure 6); when newly emerged non-treated leaves were considered, only few reads mapped to the corresponding viral region, i.e., 53 reads for the *N*-targeting dsRNA and 89 reads for *NSm*-targeting dsRNA. These results indicate that both dsRNAs are effectively introduced into the plants and processed, thus originating siRNAs that can guide the cleavage of cognate sequences. However, in agreement with the very limited amount of dsRNAs that move systemically into the plant, only few dsRNA-derived sRNAs were found in the newly emerged non-treated leaves.

The size distribution of sRNAs originating from the dsRNAs was basically uniform; in both cases, even if the amount of 21- and 22-nt reads was slightly higher than the number of other reads, no well-defined peaks related to a particular read size was observed (Figure 6). This may suggest that the inoculated dsRNA was not processed into sRNAs and the protective effect was possibly dsRNA- and not RNAi-mediated.

## Discussion

Previous work on engineering RNAi-mediated transgenic resistance indicated that constructs targeting the *N* and *NSm* genes of TSWV successfully protected plants against viral infection (Mackenzie and Ellis, 1992; Vaira et al., 1995; Prins et al., 1996; Herrero et al., 2000). Subsequent work showed that *N* gene segments as short as 110-nt were sufficient to efficiently induce RNA silencing, and hence virus resistance (Jan et al., 2000). Recently, the use of chimeric transgenes derived from different orthotospoviruses further extended the usefulness of this approach generating a broad-spectrum resistance against this virus group at the genus level (Bucher et al., 2006; Peng et al., 2014). However, even if the use of transgenic plants has been established as a powerful tool for plant protection, concerns about potential negative effects on human health and environment, together with limited acceptance by consumers dictated the development of alternative non-transgenic strategies exploiting RNAi, such as exogenous application of dsRNAs. In the present study, we tested the efficacy of the RNAi-based vaccination against the economically important virus TSWV, by applying exogenous synthetic dsRNAs on plant leaves.

We established that the RNAi-based vaccination approach is effective also against negative-strand RNA viruses, both in the model plant *N. benthamiana* and in the economically important tomato crop. However, we found that, in our conditions, the dsRNAs targeting the *N* gene are able to protect the plant, while those targeting the *NSm* gene are not. It is interesting to note that a slight reduction in the percentage of infected plants among those treated with *Rep*- and *NSm*-targeting dsRNAs was observed in respect to plants inoculated only with the virus. Such reduction is possibly related to the ability of exogenous dsRNAs to activate a sequence-unrelated pattern-induced immunity response (Niehl et al., 2016).

Overall, the different behavior of the *N*- and *NSm*-targeting dsRNAs requires some considerations.

First, we can exclude a possible effect of the dsRNA length, since the two dsRNAs are similar in size and include almost the entire coding region. Indeed, previous work on other plant viruses showed that dsRNAs with a size range from 600 to 900 nt can be very effective (Tenllado and Díaz-Ruíz, 2001).

Second, the RNAi-based vaccination has been demonstrated to be dose-dependent (Tenllado and Díaz-Ruíz, 2001; Dubrovina and Kiselev, 2019). Actually, in our experiments, the dose of dsRNAs applied to leaves (10 μg/plant) was in line with those currently used by other authors (Dubrovina and Kiselev, 2019). Furthermore, even when we increased the amount of the *NSm*-targeting dsRNAs up to 30 μg/plant, no protection against the virus was obtained (data not shown), demonstrating the intrinsic inefficacy of targeting this region.

The accessibility of dsRNAs to DCLs, frequently estimated by the abundance of originating vsRNAs, is another important aspect to consider regarding the effectiveness of RNAi-based vaccination. In our case, we can exclude that the viral sequences targeted by dsRNAs induce different response in terms of vsRNA abundance, since the amount of vsRNAs mapping to the *N* and *NSm* coding regions was similar (Mitter et al., 2013; Margaria et al., 2015; Ramesh et al., 2017). Indeed, the analysis of sRNAs populations in dsRNAs-treated plants confirmed that both *N*- and *NSm*-targeting dsRNAs were successfully recognized and processed by the endogenous RNAi machinery.

The ability of dsRNAs to move systemically in the plant can also be relevant for their efficacy. Actually, the systemic transport of exogenously applied dsRNAs is not well understood. Konakalla et al. (2016) observed that dsRNAs can move systemically in tobacco plants already 1 day following leaf application (Konakalla et al., 2016), a result also confirmed in tomato plants (Gogoi et al., 2017). Moreover, rice and maize roots soaked in a dsRNAs-containing solution can absorb dsRNAs (Li et al., 2015). On the other hand, other groups reported that exogenous dsRNAs remain mostly in treated leaves, at least in squash and watermelon plants (Kaldis et al., 2018), or in tomato (San Miguel and Scott, 2016). Such contradictory reports could be explained by the existence of an active dsRNA long-distance movement process mediated by proteins binding specific RNA-motifs (Kehr and Kragler, 2018). Our results showed that both *N*- and *NSm*-targeting dsRNAs behave similarly: a small quantity of dsRNAs can move systemically and reside in young untreated leaves already 1 day after their application on the older treated leaves, but the majority of them persist in the treated leaves. According to this, dsRNAs-derived sRNAs were mainly recovered in the leaves treated with exogenous dsRNAs and only few of them were found in newly emerged non-treated leaves. These results may suggest that, as well as dsRNAs, sRNAs also remain mostly in the treated leaves and only a small fraction is subjected to systemic transport. However, we cannot exclude the possibility that the detected sRNAs were produced in the systemic leaves after the processing of the dsRNAs coming from inoculated tissues. Taken together these data allow to rule out the possibility that the lack of protection from TSWV infection of the *NSm*-targeting dsRNA is connected with its movement ability or to the mobility of derived sRNAs.

The limited mobility of dsRNAs and derived sRNAs point out the possible limits in the large-scale use of the dsRNA-vaccination for crop protection against viruses and suggests that more effective application techniques such as the use of high-pressure spraying (Dalakouras et al., 2016) or the conjugation of dsRNAs with protein carriers (Numata et al., 2014), nanostructures (Mitter et al., 2017a), or abrasive substances could be developed.

Finally, the different antiviral efficacy of the *N*- and *NSm*-targeting dsRNAs could reside on the biological role of the *N* and *NSm* gene products. A successful infection by TSWV requires primarily the replication and transcription of the genetic viral elements to produce massive amounts of infectious ribonucleocapsid proteins (RNPs), the minimal infectious unit containing the three genomic RNAs tightly packed by the N protein and few copies of the viral RdRp. RNPs then associate with NSm and move intra- and inter-cellularly through a continuous endoplasmic reticulum network, supported by NSm-derived tubule structures that allow systemic spread (Zhu et al., 2019). Since the N protein is involved in RNPs aggregation immediately after RNA genome replication, it is likely that a certain amount of *N* coding RNA is required to synthesize enough N protein molecules to form new RNPs. Indeed, *N* gene is constitutively expressed since early time points of infection (Kormelink et al., 1994). Conversely, the *NSm* viral gene is only transiently expressed at the early stage of infection and its product is required for the RNPs to disseminate from the first initially infected cells. Therefore, one can argue that lower amount of NSm protein could be sufficient for this step. In this perspective, the degradation of the *N* coding RNA could have a crucial impact on viral replication due to the central role of its encoded protein; on the other hand, the NSm protein could play a secondary role and, even if targeted by vsRNAs, a small quantity of *NSm*-coding RNA could be sufficient to produce the required amount of movement protein. It is worth noting that, in a natural context, the inducers of antiviral RNAi and R gene-based host defense, namely dsRNAs and viral effector proteins, are deployed in the initial stages of the viral cycle (Zhu et al., 2019). The importance of the link between the biological role of *N* and NSm proteins and the ability of their encoding sequences to confer resistance is also supported by the observation that plants transformed with the *N* sequence were resistant at both plant and cellular level while plants transformed with the *NSm* sequence showed resistance at plant but not at cellular level, since the virus was still able to replicate in protoplasts of *NSm*-transgenic plants (Prins et al., 1996). However, *NSm*-transformed plants were resistant to TSWV infection probably because the *NSm* RNA was constitutively expressed in the whole plant at a level sufficient to contrast the systemic movement of the virus. Finally, it is worth mentioning that most of the successful reports on RNAi-based vaccination involved dsRNAs targeting capsid coding genes, except for one case where up to 66% of tobacco plants were protected from tobacco mosaic virus infection using dsRNAs targeting the movement protein (Sun et al., 2010).

To conclude, we demonstrated that the RNAi-based vaccination is effective also against membrane-encapsidated, multi-component, negative-sense RNA viruses and we added the *Tospoviridae*, a family including one of the most economically important plant viruses, to the list of viral families potentially targeted by this approach. Noteworthy, we showed that the choice of the viral region targeted by dsRNAs was crucial to induce resistance, highlighting that the viral cycle is a fundamental aspect to consider in the RNAi-based vaccination design.

## Data Availability Statement

All datasets generated for this study are included in the article/Supplementary Material. Sequence data are available in the Sequence Read Archive (SRA) with the BioProject ID PRJNA672300.

## Author Contributions

LM, ST, GA, AV, and EN conceived and designed the experiments. ST, MJ, and LM conducted the experiments. DM gave technical support in laboratory and greenhouse activities. SAAB supported ST activities. LM, ST, EN, GA, and AV wrote the manuscript. All authors read and approved the manuscript.

## Conflict of Interest

The authors declare that the research was conducted in the absence of any commercial or financial relationships that could be construed as a potential conflict of interest.

## Footnotes

1 http://bioinfo.ut.ee/primer3-0.4.0/

2 http://hannonlab.cshl.edu/fastx_toolkit
