# Supplementary material for: The Induction of an Effective dsRNA-Mediated Resistance Against Tomato Spotted Wilt Virus by Exogenous Application of Double-Stranded RNA Largely Depends on the Selection of the Viral RNA Target Region
Source: Front Plant Sci. 2020 Nov 26;11:533338. doi: 10.3389/fpls.2020.533338 (PMC7732615; doi:10.3389/fpls.2020.533338)
Supplement: Supplementary file 1 [file Table_1.DOCX]

| name | sequence (5’ to 3’) | purpose |
| --- | --- | --- |
| T7_TSWV_N_fw | GAATTAATACGACTCACTATAGGGAGAggttaagctcactaaggaaagca | dsRNAs synthesis |
| TSWV_N_fw | ggttaagctcactaaggaaagca | dsRNAs synthesis/  mobility |
| T7_TSWV_N_rv | GAATTAATACGACTCACTATAGGGAGAgttctgcgagttttgcctgt | dsRNAs synthesis |
| TSWV_N_rv | gttctgcgagttttgcctgt | dsRNAs synthesis/  mobility |
| T7_TSWV_NSm_fw | GAATTAATACGACTCACTATAGGGAGAggggtcttctaagtctgccag | dsRNAs synthesis/ |
| TSWV_NSm_fw | ggggtcttctaagtctgccag | dsRNAs synthesis/  mobility |
| T7_TSWV_NSm_rv | GAATTAATACGACTCACTATAGGGAGAcccttcttgtattcttggctgc | dsRNAs synthesis |
| TSWV_NSm_rv | cccttcttgtattcttggctgc | dsRNAs synthesis/  mobility |
| T7_TYLCSV_REP_fw | GAATTAATACGACTCACTATAGGGAGAtcaggtcgttttagtatcaaggct | dsRNAs synthesis |
| TYLCSV_REP_fw | tcaggtcgttttagtatcaaggct | dsRNAs synthesis |
| T7_TYLCSV_REP_rv | GAATTAATACGACTCACTATAGGGAGAcccagtgttcaagttcatcagg | dsRNAs synthesis |
| TYLCSV_REP_rv | cccagtgttcaagttcatcagg | dsRNAs synthesis |
| TSWV_L_fw | gacgacaaacacatacccgg | TSWV detection |
| TSWV_L_rv | aggagaagttaaccagcccc | TSWV detection |
| Q-TSWVN-492(+)* | tgtcttggctatatatcaggatgca | dsRNAs quantification |
| Q-TSWVN-570(-)* | taaggcttccctggtgtcatactt | dsRNAs quantification |
| qRT_TSWV_NSm_fw | tgggacagcagaaaacaataca | dsRNAs quantification |
| qRT_TSWV_NSm_rv | atcgaccagagcaaccacaa | dsRNAs quantification |
| qRT_NbCOX_fw** | cgtcgcattccagattatcca | dsRNAs quantification |
| qRT_NbCOX_rv** | caactacggatatataagrrccrraactg | dsRNAs quantification |

**SUPPLEMENTARY TABLE S1** – Primers used in this study; capital letters indicate the T7 binding site. * Mason et al. 2002; **Nerva et al. 2017
